# Examining the Type, Quality, and Content of Web-Based Information for People With Chronic Pain Interested in Spinal Cord Stimulation: Social Listening Study

**DOI:** 10.2196/48599

**Published:** 2024-01-30

**Authors:** Maarten Moens, Leen Van Doorslaer, Maxime Billot, Edgard Eeckman, Manuel Roulaud, Philippe Rigoard, Maaike Fobelets, Lisa Goudman

**Affiliations:** 1 Department of Neurosurgery Universitair Ziekenhuis Brussel Brussels Belgium; 2 STIMULUS (reSearch and TeachIng neuroModULation Uz bruSsel) Research Group Vrije Universiteit Brussel Brussels Belgium; 3 Center for Neurosciences Vrije Universiteit Brussel Brussels Belgium; 4 Department of Radiology Universitair Ziekenhuis Brussel Brussels Belgium; 5 Pain in Motion Research Group Department of Physiotherapy, Human Physiology and Anatomy Vrije Universiteit Brussel Brussels Belgium; 6 PRISMATICS (Predictive Research in Spine/Neuromodulation Management and Thoracic Innovation/Cardiac Surgery) Lab Poitiers University Hospital Poitiers France; 7 ECHO Research Group Vrije Universiteit Brussel Brussels Belgium; 8 Department of Spine Surgery & Neuromodulation Poitiers University Hospital Poitiers France; 9 Pprime Institute UPR 3346, CNRS, ISAE-ENSMA University of Poitiers Chasseneuil-du-Poitou France; 10 Biostatistics and Medical Informatics Research Group Vrije Universiteit Brussel Brussels Belgium; 11 Brussels Institute for Teacher Education Vrije Universiteit Brussel Brussels Belgium; 12 Research Foundation—Flanders (FWO) Brussels Belgium

**Keywords:** online information, social listening, neuromodulation, patient care, chronic pain, web-based data

## Abstract

**Background:**

The increased availability of web-based medical information has encouraged patients with chronic pain to seek health care information from multiple sources, such as consultation with health care providers combined with web-based information. The type and quality of information that is available on the web is very heterogeneous, in terms of content, reliability, and trustworthiness. To date, no studies have evaluated what information is available about neuromodulation on the web for patients with chronic pain.

**Objective:**

This study aims to explore the type, quality, and content of web-based information regarding spinal cord stimulation (SCS) for chronic pain that is freely available and targeted at health care consumers.

**Methods:**

The social listening tool Awario was used to search Facebook (Meta Platforms, Inc), Twitter (Twitter, Inc), YouTube (Google LLC), Instagram (Meta Platforms, Inc), blogs, and the web for suitable hits with “pain” and “neuromodulation” as keywords. Quality appraisal of the extracted information was performed using the DISCERN instrument. A thematic analysis through inductive coding was conducted.

**Results:**

The initial search identified 2174 entries, of which 630 (28.98%) entries were eventually withheld, which could be categorized as web pages, including news and blogs (114/630, 18.1%); Reddit (Reddit, Inc) posts (32/630, 5.1%); Vimeo (Vimeo, Inc) hits (38/630, 6%); or YouTube (Google LLC) hits (446/630, 70.8%). Most posts originated in the United States (519/630, 82.4%). Regarding the content of information, 66.2% (383/579) of the entries discussed (fully discussed or partially discussed) how SCS works. In total, 55.6% (322/579) of the entries did not elaborate on the fact that there may be >1 potential treatment choice and 47.7% (276/579) did not discuss the influence of SCS on the overall quality of life. The inductive coding revealed 4 main themes. The first theme of pain and the burden of pain (1274/8886, 14.34% coding references) explained about pain, pain management, individual impact of pain, and patient experiences. The second theme included neuromodulation as a treatment approach (3258/8886, 36.66% coding references), incorporating the background on neuromodulation, patient-centered care, SCS therapy, and risks. Third, several device-related aspects (1722/8886, 19.38% coding references) were presented. As a final theme, patient benefits and testimonials of treatment with SCS (2632/8886, 29.62% coding references) were revealed with subthemes regarding patient benefits, eligibility, and testimonials and expectations.

**Conclusions:**

Health care consumers have access to web-based information about SCS, where details about the surgical procedures, the type of material, working mechanisms, risks, patient expectations, testimonials, and the potential benefits of this therapy are discussed. The reliability, trustworthiness, and correctness of web-based sources should be carefully considered before automatically relying on the content.

## Introduction

### Spinal Cord Stimulation for Chronic Pain

One of the treatment options for a variety of chronic pain conditions is spinal cord stimulation (SCS), a neuromodulation technique that delivers electrical current via an implanted lead to the spinal cord [1,2]. SCS is a well-known pain management option for patients with chronic lower back pain or leg pain who are refractory to conventional management, those with complex regional pain syndrome, those with neuropathic pain syndromes, or those experiencing ischemic pain syndromes to decrease pain intensity scores and to improve the functional status of these patients [3-7]. In addition, SCS can provide greater pain relief than pharmacological treatment, improve the quality of life, and enable the return to normalized and work-related activities in some patients [8,9]. Specific to the context of persistent spinal pain syndrome and generalizable to the context of chronic pain, an interdisciplinary approach has been proposed for pain management [10,11]; therefore, several health care providers are involved in the management of these patients [12]. Most interventions in the management of chronic pain include goal setting (ie, the process of selecting treatment goals), action planning, ways of monitoring treatment progress, feedback procedures, and compliance evaluations [13,14]. Goal setting refers to the process in which goals are identified and agreed between the medical team and the patient [15]. Interviews with patients before SCS implantation revealed that all patients had goals on the level of pain reduction and walking abilities [16] and treatment expectations about improvement in the quality of life with SCS [17]. When expectations regarding SCS were explored, six themes were revealed: (1) physical well-being, (2) social well-being, (3) material well-being, (4) emotional well-being, (5) development and activity, and (6) constraints of the SCS procedure [17]. These goals and expectations are different for every patient and will highly influence patient decision-making when seeking medical help [18].

### Web-Based Health Information

In addition, as has been demonstrated in patients with other chronic diseases such as Parkinson disease, patients and their caregivers increasingly rely on the internet to gain information and access health information [19]. Previously, a gap has been identified between the availability of medical information and the translation of this knowledge to consumers of the health system [20]. Research has shown that consumers with lower back pain want clear, concise, and easy-to-understand information regarding medical intervention, physiotherapy, and other therapeutic interventions [21] and most often seek and receive information from multiple sources [20]. An increase has been documented in the number of consumers who access web-based information through web pages to search for information regarding the proposed treatments to inform personal choice. Nevertheless, there is considerable variability in the type and quality of information that is available on the web [22]. As health care professionals, it is important to be aware of the type and quality of information that is currently available to consumers on the web [23,24]. A recent study showed that approximately half of the consumers looking for health information for themselves reported that decisions regarding their treatment choices were influenced by the results of their web-based searches [22]. Moreover, web health information made resources exceedingly available and increased patients’ capabilities of influence during a consultation with a health care provider [25]. As such, the availability of web-based inquiries can complement and be used in synergy during consultations with health care providers and provide options for exploring answers to additional or forgotten questions, sensitive situations, and similar patient testimonials [26]. In contrast, quality control remains challenging, and patients’ health information literacy is highly variable [27]. Furthermore, misleading information, emotionally charged stories, and unscientific health practices are available, potentially leading to detrimental decisions in susceptible persons [26] as they experience a painful condition.

### Web-Based Information About SCS

To date, no studies have identified and evaluated what information is available over the web about SCS for patients with chronic pain. Therefore, this study applied a social listening approach, defined as an active process of attending to, observing, interpreting, and responding to a variety of stimuli through mediated, electronic, and social channels to explore web-based information about SCS [28]. The primary aim of this study was to explore the type and quality of web-based information regarding SCS for chronic pain that is freely available over the web and targeted at consumers of health care. The secondary aim was to explore the content of web-based information.

## Methods

### Search Strategy

For this study, only publicly available information from the internet was used. To collect data from the internet, a social listening tool, Awario, was consulted [29], and data were collected until July 31, 2022. Awario is a social listening software that is able to continually search various web-based platforms for keywords or phrases in new or previously made posts, with >13 billion web pages searched daily [23]. Awario used the same search term strategy as literature databases; therefore, the research question was composed according to the population, intervention, comparison, and outcome framework [30]. As keywords, we searched for “pain” and “neuromodulation” with the Boolean operator AND to refine results in 3 different languages: English, French, and Dutch. This search strategy was used to search Facebook (Meta Platforms, Inc), Twitter (Twitter, Inc, subsequently rebranded X), YouTube (Google LLC), Instagram (Meta Platforms, Inc), blogs, and the web using the following search terms for each keyword: *(“pain” OR pijn OR douleur OR #pain) AND (neurostimulation OR #neurostimulation OR neurostimulatie OR neuromodulation OR #neuromodulation OR “spinal cord stimulation” OR #spinalcordstimulation OR neuromodulatie OR ruggenmergstimulatie OR SCS OR #SCS)* to collect web-based information regarding neuromodulation for chronic pain, targeted at consumers.

### Selection Criteria

All extracted mentions were read in full by 2 independent reviewers to ensure their suitability for inclusion. This study assessed the type and quality of information available for people with chronic pain who aimed to learn more about SCS as a treatment modality. Therefore, web pages including Twitter (Twitter, Inc) posts, Facebook (Meta Platforms, Inc) posts, YouTube (Google LLC) videos, Vimeo (Vimeo, Inc) videos, Instagram (Meta Platforms, Inc) posts, blogs, and HTML web pages were included if they met the following criteria: (1) the term SCS was discussed; (2) the setting was chronic pain; and (3) the pages were written in English, French, or Dutch. Language restrictions were set for the researchers to fully comprehend the information and correctly perform quality appraisal and thematic analysis. Posts were excluded if they were not aimed at consumers (ie, patients) or were solely advertisements for specific physicians or hospitals. If discrepancies occurred between the 2 reviewers, consensus was sought through consultation and discussion with a third independent reviewer.

### Ethical Considerations

The study protocol was approved by the Ethics Committee of Universitair Ziekenhuis Brussel (B.U.N. 1432022000130) on June 22, 2022. All data used in this study are of public nature, relying on internet-based data. We did not rely on artificial intelligence to analyze the data in this study.

### Quality Appraisal and Data Extraction

After the search period, all results were extracted from Awario and imported into an Excel (Microsoft Corporation) spreadsheet to allow for data extraction. For each extracted mention, the following information was systematically extracted: location by country, type of post (eg, a web page, blog, or tweet), industry sponsorship, and information on the web page host (eg, physician, health service provider, or patient support group).

Quality appraisal of the extracted information was performed using the DISCERN instrument [31], which was specifically designed to assess written information regarding treatment choices. More specifically, the instrument provides internet users with a method to rate the quality of the obtained information [32]. The instrument consists of 15 key questions and an overall quality rating question. The first 8 questions evaluate reliability to determine whether the source of information can be trusted. The following 7 questions focus on the details of the information about the treatment options (ie, SCS in this case). The final question evaluated the overall quality rating. All questions are scored on a 5-point scale, where 1 indicates that the quality criterion has not been fulfilled and 5 indicates that the quality criterion has been completely fulfilled.

### Evidence Synthesis

All analyses were performed using the R Studio software (version 4.0.5; R Foundation of Statistical Computing). At first, a world map was created with the aid of the *rnaturalearth* package. Entries for which the country of origin could not be derived were omitted from this analysis. Entries that were created for a specific region (eg, Europe, Middle East, and Africa) were counted in the country of the headquarters of that region. NVivo Transcription (QSR International) was used to obtain the transcripts for each entry. All transcripts were screened, and manual corrections were performed where necessary. A thematic analysis was performed in NVivo 12 (QSR International), in which data were categorized under different themes and subthemes through inductive coding according to standard thematic analysis guidelines. The applied thematic analysis consisted of familiarization, coding sentences, categorizing codes, and developing themes and subthemes. During the first cycle of coding, mostly descriptive and holistic coding methods were used, and to a minor degree, structural coding methods were assessed. During the second cycle of coding, a focused coding method was used to develop themes and subthemes. A word cloud of the most frequently used words was calculated after removing common stop words (eg, slang, abbreviations, or ironic unconventional written expressions).

## Results

### Search Results

**Figure 1:**
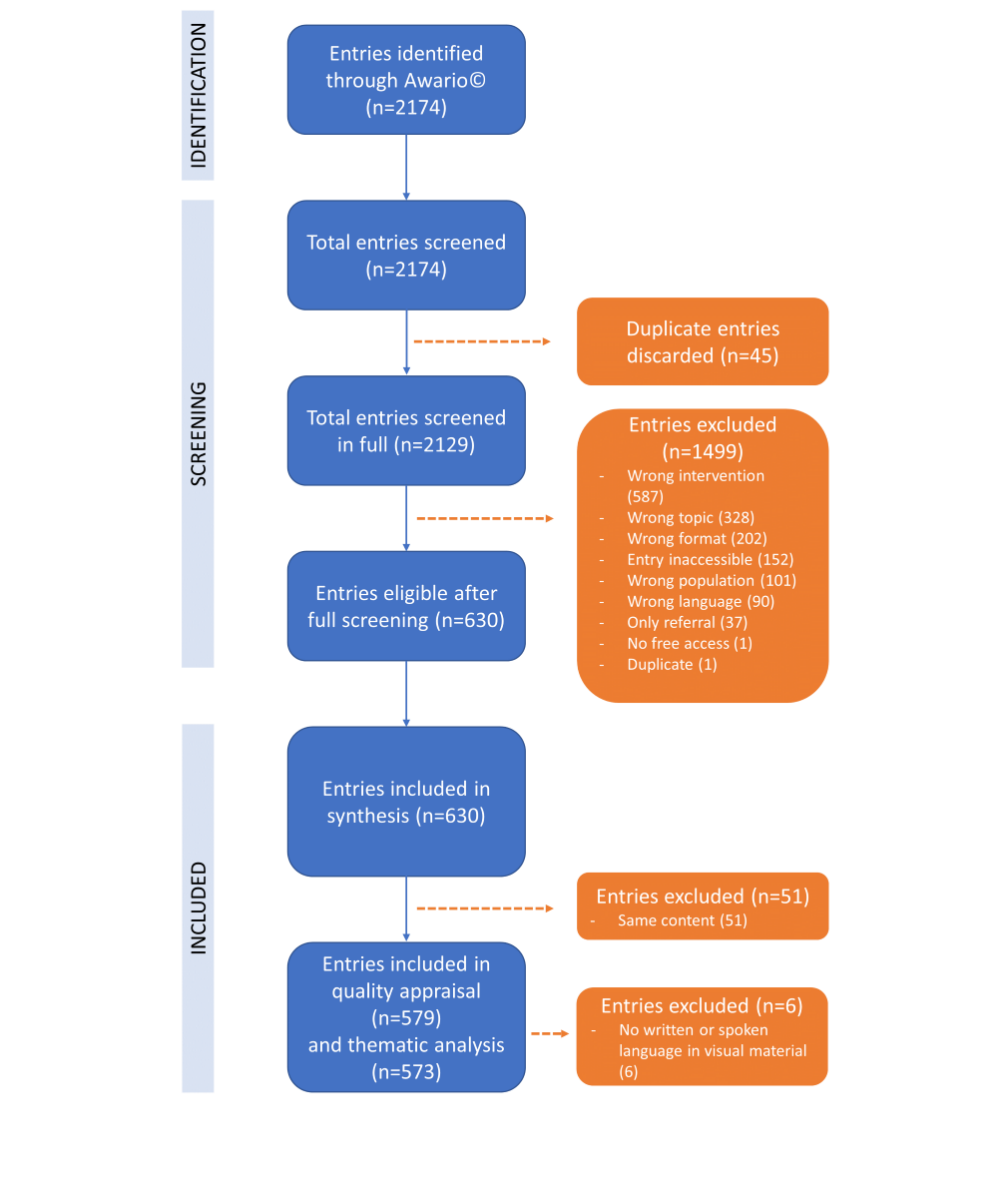
Flowchart representing the screening process for this study in which web-based information about spinal cord stimulation (SCS) for chronic pain was retrieved with a social listening design. Blue boxes indicate the flow of included entries, whereas orange boxes give indications on excluded entries.

Using Awario, we identified 2174 web-based entries, of which 45 (2.07%) duplicate entries could be removed immediately. The remaining 2129 (97.93%) entries were evaluated by 2 reviewers independently, where the percentage of agreement between both reviewers for full-entry screening was 93.85%. After this full-entry screening of 2129 entries, 1 (0.05%) entry was excluded because there was no free access, 587 (27.57%) were excluded because of wrong intervention (no SCS), 37 (1.74%) were excluded because these only consisted of a referral to a specific center or physician or referral to SCS without explanation about the treatment, and 101 (4.74%) were excluded because of wrong population. Of 2129 entries, the format was not applied to patients for 202 (9.45%) entries, and 328 (15.41%) entries were excluded owing to the wrong topic that was discussed. In addition, 152 (7.14%) entries were inaccessible, and 90 (4.23%) were excluded based on language. One additional duplicate was found during the full screening. Figure 1 presents the flowchart of this study. Thus, after screening, 1499 (70.41%) entries were excluded and 630 (29.59%) were included in this study. For quality appraisal and thematic analysis, entries with a different URL address but with the same content as another entry were removed from this analysis. Therefore, quality appraisal was performed for 579 (27.19%) entries. Within the thematic analysis, 6 entries were excluded because of the lack of spoken or written material.

**Figure 2:**
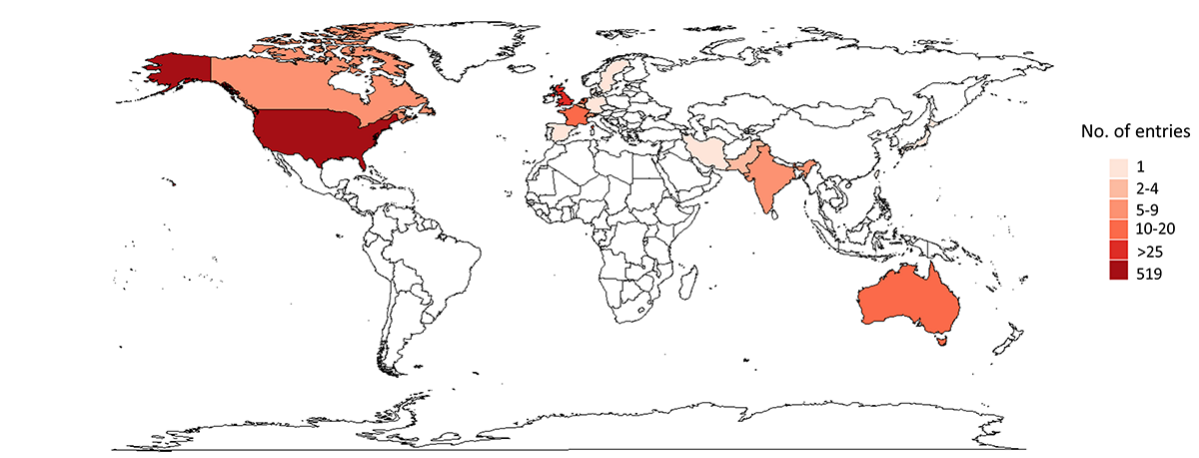
World map indicating the country of origin of the included web-based entries that were available about spinal cord stimulation for chronic pain. Darker red color indicates a higher count of entries. White color indicates that no entries originated from that respective country. For 10 entries, no country could be derived; therefore, 620 entries are represented on this world map.

The included 630 web-based entries could be categorized as web pages, including news and blogs (114/630, 18.1%); Reddit (Reddit, Inc) posts (32/630, 5.1%); Vimeo (Vimeo, Inc) hits (38/630, 6%); or YouTube (Google LLC) hits (446/630, 70.8%). The upload date of the entries ranged from October 30, 2007, to July 27, 2022, with the median date on November 1, 2019 (quartile 1 to quartile 3: June 11, 2016, to July 8, 2021). The entries were created by institutions (ie, medical centers, expert institutes, expert societies, and universities; 311/630, 49.4%), industry (133/630, 21.1%), patients (73/630, 11.6%), health care providers (49/630, 7.8%), television shows (43/630, 6.8%), and others (21/630, 3.3%). Figure 2 presents the country of origin for all entries on the world map. Most posts originated in the United States (519/630, 82.4%). Furthermore, posts originated in the following countries: United Kingdom (26/630, 4.1%), the Netherlands (16/630, 2.5%), France (11/630, 1.8%), Australia (11/630, 1.8%), Canada (9/630, 1.4%), and India (7/630, 1.1%). To a lesser degree, posts originated in Switzerland, Belgium, Germany, Denmark, Spain, Ireland, Japan, Luxembourg, Pakistan, Sweden, Singapore, and Taiwan. For 10 (1.6%) out of 630 entries, the location could not be derived. Of 630 entries, 274 (43.5%) were created by an industrial partner of neuromodulation devices or denoted a specific industry partner, whereas 356 (56.5%) were not associated with an industrial partner. World maps and entry types based on industry-related and non–industry-related entries are provided in Multimedia Appendix 1. World maps presenting the locations of the 4 prominent companies using neuromodulation devices are provided in Multimedia Appendix 2.

In Multimedia Appendix 3, the word cloud is presented (80 most frequent words) with a clear dominance of the word “pain.” As most of the entries were written in English, with few articles in French or Dutch, the most frequently stated terms were in English.

### Quality Appraisal

Regarding the content of information, 66.2% (383/579) of the entries discussed (at least partially) how SCS works. The risks of treatment were only discussed in a limited number of entries (142/579, 24.5%). In total, 55.6% (322/579) of the entries did not elaborate on the fact that there may be >1 potential treatment choice and 47.7% (276/579) did not discuss the influence of SCS on the overall quality of life. Regarding the reliability of information, 72.2% (418/579) of the entries did not clearly describe the aims that they aimed to achieve. In 94.8% (549/579) of the entries, it was clear or partially clear when the information in the entry was produced. Areas of uncertainty were not discussed in most entries (313/579, 54.1%). The overall quality of entries was 2.5 on a scale of 5, in which Reddit (Reddit, Inc) posts scored 2.6, Vimeo (Vimeo, Inc) videos 2.5, web pages 2.8, and YouTube (Google LLC) hits 2.4 out of 5, respectively. The complete quality appraisal, including the number of entries that did not fulfill the quality criteria (score 1), partially fulfilled the quality criteria (scores 2-4), or completely fulfilled the quality criteria (score 5), for each question of the DISCERN instrument is presented in Table 1. In addition, the average scores are shown for all entries and separated by the type of entry.

**Table 1:** Quality appraisal scoring of the available web-based information concerning spinal cord stimulation for chronic pain using the DISCERN instrument (N=579).

Category and questions		No^a^ (score 1), n (%)	Partially^b^ (scores 2-4), n (%)	Yes^c^ (score 5), n (%)	Total (N=579), mean (SD)	Reddit (Reddit, Inc) posts (n=32), mean (SD)	Vimeo (Vimeo, Inc) videos (n=35), mean (SD)	Web pages (n=105), mean (SD)	YouTube (Google LLC) hits (n=407), mean (SD)
**Reliability**									
	Are the aims clear?	*418 (72.2)* ^d^	134 (23.1)	27 (4.7)	1.6 (1.1)	1.9 (1.0)	1.5 (1.0)	2.2 (1.4)	1.5 (1.0)
	Does the publication achieve its aims?^e^	11 (6.8)	*134 (83.2)*	16 (9.9)	3.1 (0.9)	3.1 (0.3)	3.0 (0.0)	3.3 (1.2)	3.0 (0.8)
	Is the publication relevant?	70 (12.1)	*416 (71.9)*	93 (16.1)	3.0 (1.2)	3.6 (1.2)	3.6 (0.9)	3.7 (1.2)	2.7 (1.0)
	Is it clear what sources of information were used to compile the publication?	105 (18.1)	*414 (71.5)*	60 (10.4)	2.9 (1.1)	2.8 (0.8)	3.0 (1.0)	2.7 (1.6)	2.9 (0.9)
	Is it clear when the information used or reported in the publication was produced?	30 (5.2)	*502 (86.7)*	47 (8.1)	3.1 (0.7)	3.1 (0.3)	3.2 (0.6)	3.1 (1.1)	3.1 (0.7)
	Is the publication balanced and unbiased?	15 (2.6)	243 (41.9)	*321 (55.4)*	4.1 (1.1)	4.1 (1.3)	4.5 (0.9)	4.3 (1.0)	4.1 (1.1)
	Does the publication provide details of additional sources of support and information?	215 (37.1)	*325 (56.1)*	39 (6.7)	2.4 (1.2)	1.3 (0.9)	2.3 (1.1)	2.9 (1.5)	2.4 (1.0)
	Does the publication refer to areas of uncertainty?	*313 (54.1)*	103 (17.8)	163 (28.2)	2.5 (1.7)	3.8 (1.6)	1.9 (1.5)	2.6 (1.7)	2.4 (1.8)
**Content of information**									
	Does the publication describe how each treatment works?	196 (33.9)	146 (25.2)	*237 (40.9)*	3.1 (1.7)	1.2 (0.8)	2.5 (1.7)	3.9 (1.5)	3.2 (1.7)
	Does the publication describe the benefits of each treatment?	*267 (46.1)*	253 (43.7)	59 (10.2)	2.2 (1.3)	1.3 (0.7)	2.2 (1.1)	2.5 (1.5)	2.3 (1.3)
	Does the publication describe the risks of each treatment?	*437 (75.5)*	89 (15.4)	53 (9.2)	1.7 (1.3)	1.8 (1.4)	1.7 (1.2)	2.2 (1.6)	1.5 (1.1)
	Does the publication describe what would happen if no treatment is used?	*540 (93.3)*	30 (5.2)	9 (1.6)	1.2 (0.7)	1.9 (1.5)	1.2 (0.6)	1.1 (0.6)	1.1 (0.5)
	Does the publication describe how the treatment choices affect overall quality of life?	*276 (47.7)*	99 (17.1)	204 (35.2)	2.7 (1.8)	2.8 (1.7)	2.9 (1.9)	2.1 (1.6)	2.9 (1.8)
	Is it clear that there may be >1 possible treatment choice?	*322 (55.6)*	119 (20.6)	138 (23.8)	2.4 (1.7)	3.3 (1.7)	2.2 (1.5)	3.1 (1.8)	2.1 (1.6)
	Does the publication provide support for shared decision-making?	*316 (54.6)*	139 (24)	124 (21.4)	2.3 (1.6)	3.6 (1.5)	2.7 (1.7)	2.3 (1.6)	2.2 (1.6)
**Overall quality**									
	On the basis of the answers to all of the above questions, rate the overall quality of the publication as a source of information about treatment choices	9 (1.6)	*570 (98.5)*	0 (0)	2.5 (0.6)	2.6 (0.6)	2.5 (0.5)	2.8 (0.7)	2.4 (0.6)

^a^Entries that did not fulfill the quality criteria.

^b^Entries that partially fulfilled the quality criteria.

^c^Entries that completely fulfilled the quality criteria.

^d^Italicization indicate the most frequent answer.

^e^This item was not scored if item 1 was scored as no.

### Thematic Analysis

#### Overview

During inductive coding, four main themes were revealed: (1) pain and the burden of pain with 14.34% (1274/8886) of coding references, (2) neuromodulation as a treatment approach with 36.66% (3258/8886) of coding references, (3) device-related aspects with 19.38% (1722/8886) of coding references, and (4) patient benefits and testimonials of treatment with SCS with 29.62% (2632/8886) of coding references.

#### Pain and the Burden of Pain

In the first theme, pain and the burden of pain were described with 14.34% (1274/8886) of coding references from 56.7% (325/573) of coded entries. The first subtheme discussed pain and chronic pain with information on pain awareness, how to objectify pain, the definition of chronic pain, the duration of pain, potential causes of pain, the incidence of pain, a closer look at neuropathic pain, other types of pain, and the heterogeneity of pain. The second subtheme elaborated on other pain management techniques compared with SCS in which pain medication, epidural infiltrations or nerve blocks, spinal surgery, physical therapy, psychological therapy, or even a combination of techniques were described. The third subtheme evaluated how pain can affect an individual, with a focus on symptoms of pain, the misinterpretation of input signals as a painful experience during chronic pain, and potential implications on social life, daily living, bodily functions, and emotional distress. The final subtheme reported on patient experiences and stories of living with chronic pain. Within this subtheme, individual stories about pain physicians and quotes on how chronic pain feels are discussed. In addition, the burden of the time delay in finding the right help and the misunderstanding of other people who do not understand pain were categorized under this subtheme.

#### Neuromodulation as Treatment Approach

**Figure 3:**
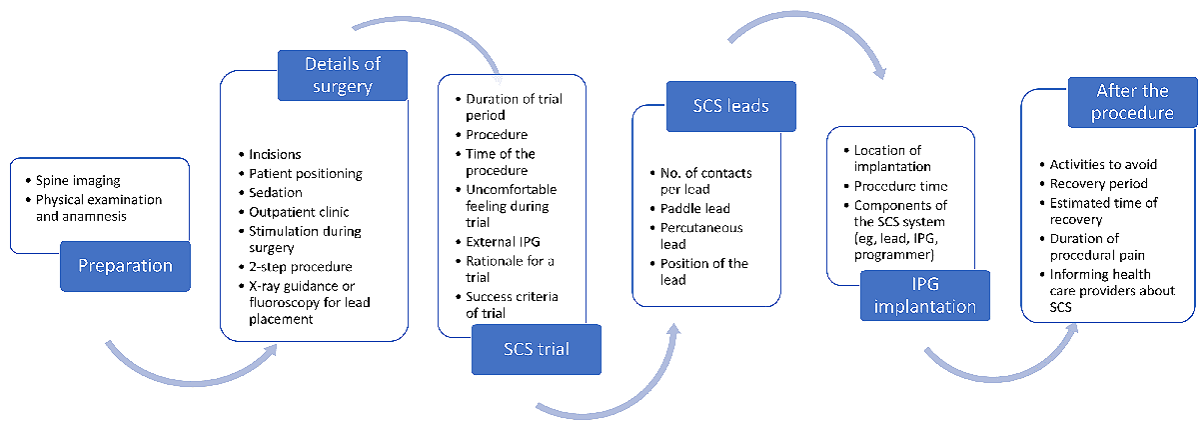
Identified topics about the spinal cord stimulation (SCS) procedure for chronic pain for which web-based information is available, as identified during the thematic analysis. IPG: implantable pulse generator.

In the second theme, information about neuromodulation, in general, was pooled with 36.66% (3258/8886) of coding references from 86.9% (498/573) of coded entries. The first subtheme that was identified focused on access to neuromodulation, different techniques, targets of neuromodulation, limitations of these therapy options, history, underlying working mechanisms (eg, gate control theory), and the fact that neuromodulation masks pain and does not heal it. Patient-centered care was revealed as the second subtheme, in which a multidisciplinary approach including patient empowerment, independence, and education, with a holistic evaluation and shared decision-making with physicians and family members was discussed. Another subtheme consisted of SCS therapy, in which the definition of SCS was stated and the uncertainties with response to success rate (eg, trial pain relief vs pain relief after implantable pulse generator [IPG], responder rates, SCS outcome variables, and patient selection) and the combination of multiple neuromodulation devices was discussed. Furthermore, the SCS procedure itself was classified as a distinct subtheme and is presented in Figure 3. The final subtheme discussed the risks of the treatment (Table 2).

**Table 2:** Reported risks of spinal cord stimulation (SCS) for chronic pain that were revealed during the thematic analysis of the available web-based information. Counts present the number of entries that discussed each risk of SCS compared with the number of web-based entries in the thematic analysis (N=573).

Topic and risks of SCS		Frequency, n (%)
**Pain**		
	Additional pain after procedure	2 (0.4)
	Inadequate pain relief or loss of therapy effect	48 (8.4)
**Adverse events and serious adverse events**		
	Allergic reactions	13 (2.3)
	Bleeding	20 (3.5)
	Dural puncture	4 (0.7)
	Fluid accumulation	6 (1.1)
	Scar tissue	7 (1.2)
	Headache	7 (1.2)
	Infection	77 (13.4)
	Injuries to spinal cord	7 (1.2)
	Nerve injury	13 (2.3)
	Neurological deficit	5 (0.9)
	Paralysis	21 (3.7)
	Poor wound healing	1 (0.2)
	Reaction to anesthetic agent	1 (0.2)
	Skin erosion	1 (0.2)
	Soreness or bruising	3 (0.5)
	Mental distress	1 (0.2)
	Death or injury	2 (0.4)
**Device-related risks**		
	Positional sensitivity (ie, distance between electrode and spinal cord)	1 (0.2)
	Hardware issues	22 (3.8)
	Fracture of electrode	4 (0.7)
	Electromagnetic interference	26 (4.5)
	Disconnection electrode–IPG^a^	5 (0.9)
	Lead migration	68 (11.9)
	Pain at implantation site	34 (5.9)
	Uncomfortable stimulation	13 (2.3)
**General risks**		
	Complications are extremely rare	7 (1.2)

^a^IPG: implantable pulse generator.

#### Device-Related Aspects

In the third theme, device-related aspects were discussed (1722/8886, 19.38% of coding references from 379/573, 66.1% of coded entries), in which the charger and remote programming, the different device manufacturers of SCS, disadvantages of SCS, innovations, programming possibilities, types of stimulation, and scientific studies were included. Specifically focusing on the potential disadvantages of SCS, the following aspects were noted: difficulties with charging the IPG, magnetic resonance imaging (MRI) restrictions, restrictions with driving and operating machinery, insufficient coverage of the targeted region, implications on other medical procedures owing to the presence of SCS device, the need for remote control (ie, the lack of control through mobile phone), replacement of nonrechargeable IPG, detection of the SCS system at security gates, the potential of nausea, and the therapy burden in general (eg, need for a registration card and recharging process). The innovative aspects highlighted were as follows: accelerometry, closed-loop stimulation, constantly evolving technology, improved battery life, increased competition in the market, increased market value of SCS manufacturing companies, MRI safety, the possibility of multiple waveform programming, new waveforms and software, more precise stimulation targets (ie, better coverage), rechargeable and nonrechargeable devices, running multiple SCS stimulation programs simultaneously, x-ray and computed tomography safety, and various sizes of IPGs.

#### Patient Benefits and Testimonials of Treatment With SCS

**Figure 4:**
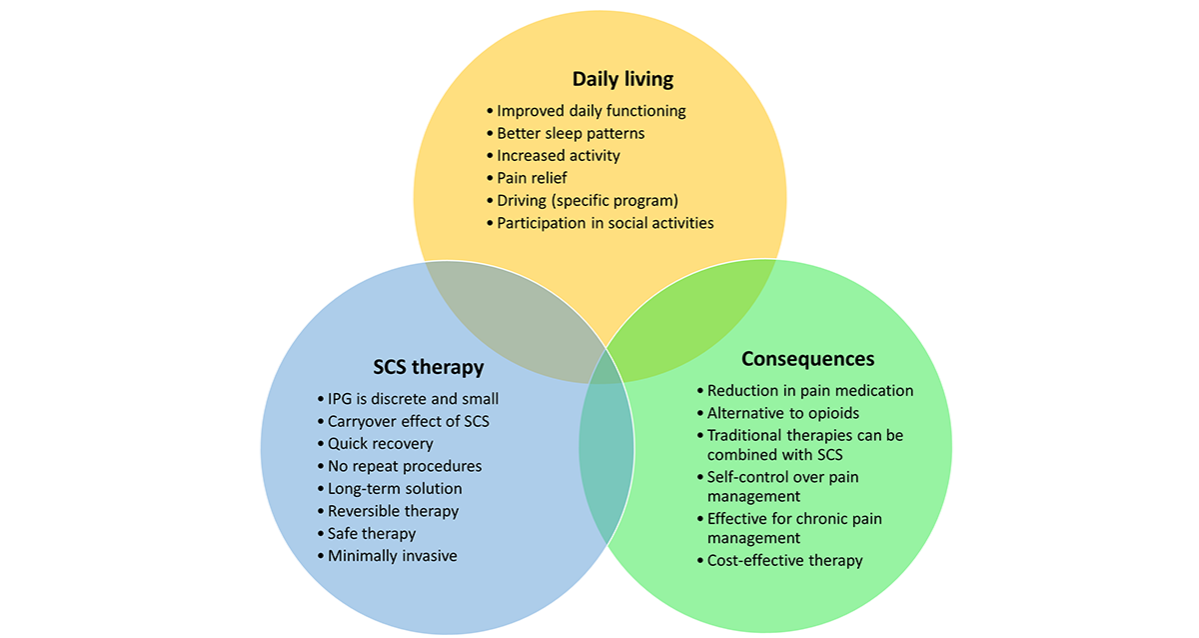
Benefits of a treatment trajectory with spinal cord stimulation (SCS) for chronic pain that were found during the thematic analysis of the web-based information. IPG: implantable pulse generator.

In the last theme (2632/8886, 29.62% of coding references from 514/573, 89.7% of coded entries), 3 subthemes were identified: benefits of SCS through patient experiences, candidates for SCS, and patient testimonials. The benefits of SCS that were reported are presented in Figure 4. The most frequently stated aspects were alternatives to opioids, pain relief, improved daily functioning, reduction in pain medication use, reversible therapy, and a minimally invasive approach. In the second subtheme about patient selection and candidate selection, contraindications were identified, including candidate checklists, insurance coverage, pain location, conditions that are suitable for SCS, pain description, pain relief during the trial period, psychological screening, selection process, tobacco use, and SCS as the last resort therapy. In the final subtheme, patient testimonials were combined, in which doubts about stimulation, expectations before SCS, the exchange of experiences and information, SCS coverage, living with an implanted device, pain scores, postoperative pain, and patient goals were discussed. Finally, the positive and negative experiences reported in the testimonials are presented in Textbox 1.

Table 3 presents a selection of quotes for each theme. Table 4 presents the frequency of themes and subthemes.

**Negative and positive experiences with spinal cord stimulation for chronic pain that were revealed during thematic analysis in patient testimonials. Counts present the number of entries that expressed these experiences.**

**Negative experiences**

- Adjustments in stimulation are difficult (n=1)
- Lack of follow-up after surgery by physicians (n=2)
- Negative patient experiences (n=5)
- Negative experiences with insurance or compensation (n=6)
- Negative thoughts on spinal cord stimulation by patients who were never implanted (n=5)
- Reduced trust in health care (n=10)
- Wrong stimulation location (n=2)

**Positive experiences**

- Increase in activities of daily living (n=50)
- Increase in functionality (n=25)
- Return to work (n=7)
- Life-changing therapy (n=73)

**Table 3:** A selection of quotes that was revealed during the thematic analysis of available web-based information about spinal cord stimulation (SCS) for chronic pain.

Theme, subtheme, and context				Quote
**Pain and the burden of pain**				
	**Pain and chronic pain**			
		Definition of chronic pain presented by a health care provider.	“Chronic pain as pain that persists beyond the expected recovery period or pain that accompanies a chronic health condition. Because this pain is not productive and is not the result of an ongoing injury, it is referred to as pathological and is therefore treated as a condition, not just as a symptom.”	
	**How pain can affect an individual**			
		Penny, a woman who had pain for >10 years explained how the pain impacted her functioning.	“I rarely left the house. I could only just cope with the very basic way of living. I just really felt like I wanted to give up. It was just all too much.”	
	**Patient experiences and stories on living with chronic pain**			
		While reviewing her SCS system, Sarah explained how chronic pain makes her feel.	“But I mean, there’s been lots of times that I have locked myself in my room and cried all night just because I couldn’t imagine dealing with this pain one more hour. Obviously, it’s not like that every day, but sometimes it is.”	
**Neuromodulation as a treatment approach**				
	**Patient-centered care**			
		Soula discussed about her involvement in her treatment and working together with the company representatives.	“And of course, I can have as many programs put on it. I basically work it out together with the programmers. Recently, for example, I went back and said: ‘ah my feet are really sore. I really need more stimulation on my feet.’ And so I had two new programs popped on there, and they’ve been fantastic.”	
		Furthermore, Soula elaborated on her independence as a patient.	“That, to me, makes me feel I’ve got options of long term options that I don’t have these big health appointment calendar that I have to meet and that all the things that I need for my management are tucked into my body look like who they are. You then have to go anywhere or reach for anything to some difficult. It really is just in me that makes me feel quite independent, makes me feel really strong about the way that I manage day to day.”	
**Device-related aspects**				
	**Potential disadvantages**			
		Reddit (Reddit, Inc) video	“Keep that thing charged. I’ve noticed the closer I get to empty, the less effective it is.”	
		Pam discussed these elements in her video.	“Remember, it is a life changing experience in more than one way. It may help you a great deal. But the other issue that you have to deal with is having something implanted inside you that you that you have to carry a card to make sure that if you ever have a medical procedure like an MRI, that it’s understood what kind of device you have and can you have an MRI if you travel, if you go off on a weekend, you have to be able to charge this device so you’re tethered to a device.”	
	**Innovative aspects**			
		Example from a physician and researcher	“There is no limit for these indications, this is why I love neuromodulation, it’s an evolving field and every six months at the latest we have something new not only in the spinal cord stimulation but also in all other options.”	
**Patient benefits and testimonials of treatment with SCS**				
	**Patient testimonials**			
		Eric’s quote expressed a positive feeling.	“The way I’m living right now, if you would have known me five or six years ago, you would have never guessed. Before I couldn’t walk from my front room to the kitchen and now running marathons and enjoying holiday company from everybody else.”	
		Tom’s quote represented some doubts.	“At first, I was a little reluctant. I didn’t know how I would feel about something I was going to be, you know, inserted into my body. Is too much, is it too fast? That I realized that after the last 47 years, I really didn’t have anything to risk. I wanted to feel better.”	
		A Reddit (Reddit, Inc) post expressed a negative experience.	“So... Now it’s been 3 months since surgery. It’s not even coming close to relieving any pain.”	

**Table 4:** Frequency of themes and subthemes identified in web-based information about spinal cord stimulation (SCS) for chronic pain during the thematic analysis expressed by aggregated coding references (N=8886) and aggregated coded entries (n=573).

Themes and subthemes			Coding references, n (%)		Coded entries, n (%)
**Pain and the burden of pain**			1274 (14.3)		325 (56.7)
	Pain and chronic pain	230 (2.6)		125 (21.8)	
	Pain management techniques	601 (6.8)		218 (38)	
	How pain can affect an individual	398 (4.5)		183 (31.9)	
	Patient experiences and stories on living with chronic pain	45 (0.5)		36 (6.3)	
**Neuromodulation as a treatment approach**			3258 (36.7)		498 (86.9)
	Neuromodulation	513 (5.8)		201 (35.1)	
	Patient-centered care	345 (3.9)		195 (34)	
	SCS therapy	480 (5.4)		303 (52.9)	
	SCS procedure	1437 (16.2)		320 (55.8)	
	Risks of the treatment	483 (5.4)		127 (22.2)	
**Device-related aspects**			1722 (19.4)		379 (66.1)
	Charger and remote programming	109 (1.2)		76 (13.3)	
	Different device manufacturers for SCS	190 (2.1)		113 (19.7)	
	Disadvantages of SCS	157 (1.8)		95 (16.6)	
	Innovations	554 (6.2)		208 (36.3)	
	Programming possibilities	220 (2.5)		117 (20.4)	
	Type of stimulation	407 (4.6)		200 (34.9)	
	Scientific studies	85 (1)		40 (7)	
**Patient benefits and testimonials of a treatment with SCS**			2632 (29.6)		514 (89.7)
	Benefits of SCS through patient experiences	1148 (12.9)		376 (65.6)	
	Candidates for SCS	1109 (12.5)		365 (63.7)	
	Patient testimonials	375 (4.2)		187 (32.6)	

## Discussion

### Principal Findings

Patient-centered care, described as shared decision-making through patient autonomy, has drastically increased during the last decade [33]. Medical and health information contribute to equalizing the balance of power between patients and health care providers and thus to patient empowerment [25]. Through this approach, patients are supported to make informed and active choices concerning their health, compared with fulfilling the role of a passive recipient of care [34]. Empowered decision-making in treatment selection is inevitably related to appropriate communication and information tools and greater patient involvement in the decision-making process [35]. Therefore, this study evaluated the availability and content of information on the internet regarding SCS, which is a crucial prerequisite to enhance empowered decision-making in the field of neuromodulation.

The results of this study clearly indicated that most of the information originated in the United States (519/630, 82.4%). Besides the dominance of the United States, posts also originated in the United Kingdom (26/630, 4.1%), the Netherlands (16/630, 2.5%), France (11/630, 1.8%), Australia (11/630, 1.8%), Canada (9/630, 1.4%), and India (7/630, 1.1%). None of the included entries could be linked back to Africa or South America, potentially because of the language restrictions. The field of neuromodulation is driven by technical innovations by device manufacturers, with company headquarters located in the United States. This may be one of the potential explanations for the dominance of information in this region.

During thematic analysis, 4 major themes were identified, namely pain, neuromodulation as a treatment approach, device-related aspects, and patient benefits and testimonials. In these themes, important aspects in the field of neuromodulation were discussed, from patient selection, over the SCS trial period and IPG implantation, implanted materials, risks, benefits, and expectations to the place of neuromodulation within the treatment arsenal of patients with chronic pain. There were no gaps identified regarding crucial information that was lacking. The content of information that is available for patients is closely aligned with the latest research developments, for example, continued technological advancements [36], holistic evaluations [37], and ethical considerations, such as MRI conditional devices [38]. This indicates that recent research developments have been timely translated into layperson terms to make them available to the public, potentially because of the strong valorization of project plans and effective marketing strategies, in contrast to the lack of or limited knowledge translation that is often observed [39,40]. Nevertheless, the availability of web-based information does not mean that this information is easily accessible to health care consumers. Previous research on patients with persistent spinal pain syndrome type 2 indicated that 85.3% had a low position in the social hierarchy (ie, a low social gradient of health) [41]. On the basis of qualitative research, patients are particularly interested in having information in layperson terms with concise language, contextualization of values, and definitions of medical terms to increase their understanding [42]. Nevertheless, in health care, it is rare to find a layperson knowledgeable in medical terminology, potentially leading to poor understanding of the medical conditions and potential treatments [43]. This emphasizes the need for layperson terms to ensure that populations with a low position in the social hierarchy have equal opportunities to enhance patient empowerment. Especially in the current digital era, patients develop toward so-called e-patients and are more prone to obtaining complementary web-based health information. Nevertheless, this evolution may give rise to larger interindividual differences based on socioeconomic status in cases where patients do not have access to health resources, eventually creating less-informed patients and, as such, enlarging disparities. In addition, the availability of information does not imply that information is centralized; therefore, real-time capturing of the web-based information-seeking process should be conducted [44]. This advocates further evaluation of the web-based information-seeking process to determine whether patients have access to information and whether the information is effectively understandable by the target population.

Besides an evaluation of the availability of information, the provided information should also be reliable to trust the obtained information. The DISCERN instrument was used to conduct a quality appraisal [45], where poor scores were mainly because of a lower scoring on the content of information than on the reliability. Overall, information was deemed reliable, where creators of new content should primarily focus on clearly defining the aims of their posts, videos, or web pages and the areas of uncertainty to further enhance the reliability of web-based information. During the evaluation of the content, the broader perspective of the therapeutic intervention was explored (ie, alternative treatment options, risks of every treatment option, what if treatment is not applied, and shared decision-making). Neuromodulation techniques, including SCS, are often considered a last resort therapy after all other pain management procedures fail to alleviate the pain. It may be hypothesized that alternative treatments and risks, quality of life, and benefits of other treatments were less frequently discussed as SCS is a final pain management (ie, pain control) option and not a therapeutic (ie, healing) option, where several techniques could be applied to treat a specific condition. Nevertheless, despite the poor scoring of many entries on the content of information, there are sources where the information is available because the results of the content analysis could not identify an obvious knowledge gap. Combining these results, the field may benefit from more centralized pages or complete overviews of neuromodulation techniques in perspective to the complete therapeutic arsenal of pain management techniques.

The relationship among health care providers, patients, and industrial device representatives is a *conditio sine qua non* in the field of neuromodulation. Despite the disputable role of industry-sponsored randomized controlled trials of drugs and medical devices toward more favorable results compared with non–industry-sponsored trials [46] and the influence of medical promotional tools provided by medical representatives on physicians’ prescribing practices [47], in total, 43.5% of the retrieved information referred to a specific industrial partner (directly or indirectly). Notwithstanding the considerable involvement of device manufacturers, web-based information is not limited to commercial information about the different types of materials and IPGs, as revealed by the heterogeneity of detected themes and subthemes in the content analysis. This clearly indicates that there is a difference in bias introduced by industry funding versus information that is available to the public.

This study indicated that a lot of information about SCS is available on the web, where it should be clearly stated that this information only provides an additional resource for patients besides the traditional health care system and consultation with multidisciplinary pain management teams. While interpreting these results, a couple of limitations should be considered. Notably, only web-based information that was provided in English, French, or Dutch was retrieved from our search. In addition, web-based information that is spread through LinkedIn (Microsoft Corporation) was underrepresented because information retrieval from LinkedIn is not fully supported owing to the limitations of the official application programming interface (retrieved through personal communication with an Awario representative). In addition, social listening only reflects the narratives and discourse occurring in public, internet-based spaces and may not mirror information from closed platforms and non–internet-based channels [48]. Finally, within the search strategy, the most common synonyms of SCS were listed; however, materials that extend beyond the terms used will not have been retracted with the current search strategy.

### Conclusions

This social listening study regarding the availability of web-based information about SCS demonstrated that health care consumers have the possibility to access web-based information about SCS, including details about the surgical procedures, type of material, working mechanisms, risks, patient expectations, testimonials, and the potential benefits of this therapy option. The availability of information, however, does not evaluate whether information is effectively retrieved by consumers of health care and whether it is easily accessible, which needs to be further evaluated. In addition, quality control of web-based information is extremely difficult, and therefore, reliability, trustworthiness, and correctness of sources should be carefully considered before automatically relying on the content of the sources.

## Appendix

Multimedia Appendix 1 World maps and entry types for industry-related and non–industry-related entries.

Multimedia Appendix 2 World maps present the locations of the 4 prominent companies using neuromodulation devices.

Multimedia Appendix 3 Word cloud based on the transcripts of the included entries from the social listening study about spinal cord stimulation for chronic pain.

## Abbreviations

IPG: implantable pulse generator
MRI: magnetic resonance imaging
SCS: spinal cord stimulation

## Data Availability

The data set generated and analyzed during this study is available from the corresponding author upon reasonable request.
