# Supplementary material for: Examining the Type, Quality, and Content of Web-Based Information for People With Chronic Pain Interested in Spinal Cord Stimulation: Social Listening Study
Source: J Med Internet Res. 2024 Jan 30;26:e48599. doi: 10.2196/48599 (PMC10865187; doi:10.2196/48599)
Supplement: Multimedia Appendix 1 [file jmir_v26i1e48599_app1.docx]

**Supplementary Material I**


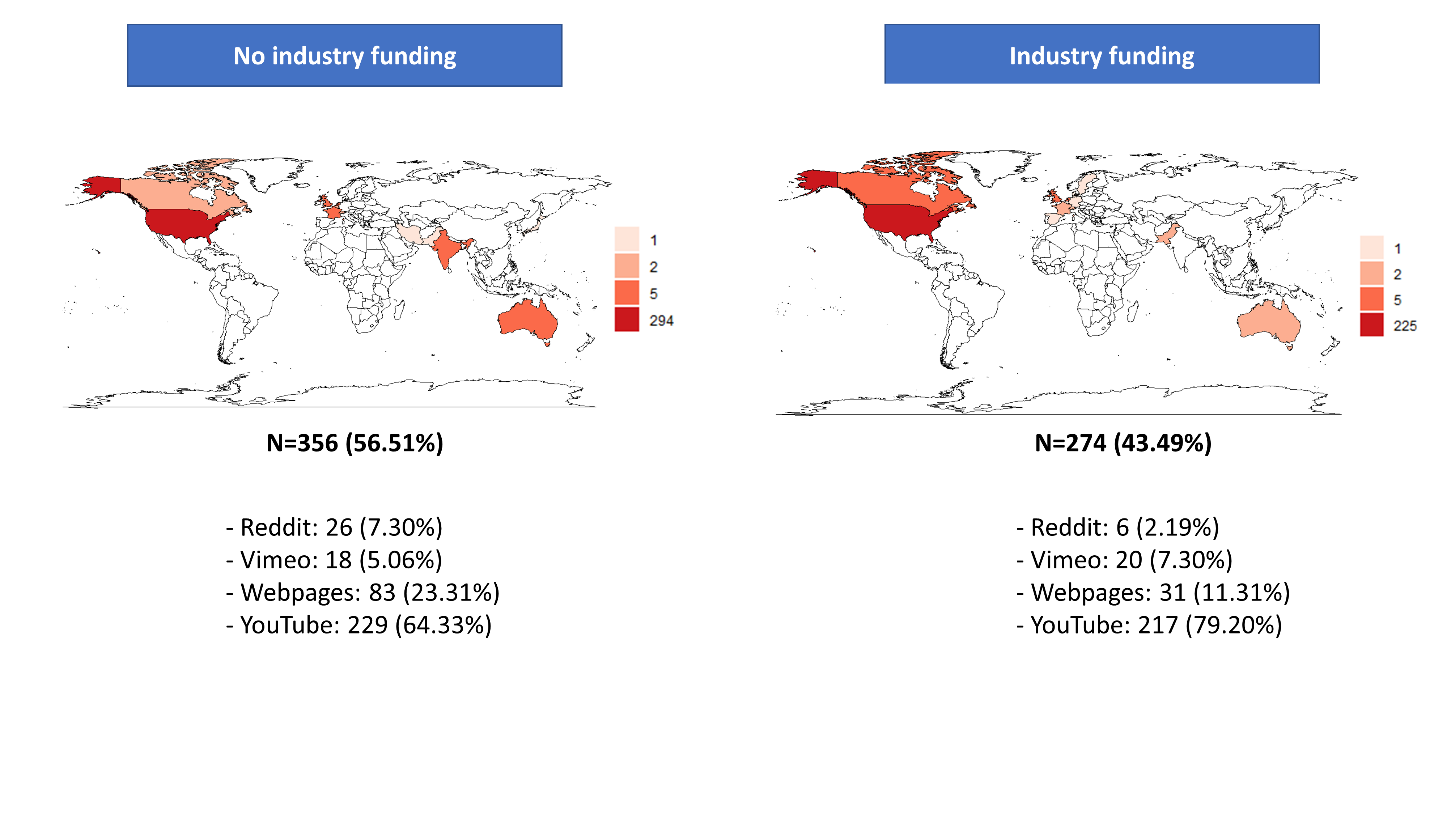


World map denoting the country of origin of the included web-based entries, separated by industry involvement. Entries were counted as industry related in case an industrial partner of neuromodulation devices constructed the entry or in case a specific industry partner was denoted in the entry. Darker red colours indicate a higher count. A white colour indicates that no entries originated from that respective country.
