# Supplementary material for: Examining the Type, Quality, and Content of Web-Based Information for People With Chronic Pain Interested in Spinal Cord Stimulation: Social Listening Study
Source: J Med Internet Res. 2024 Jan 30;26:e48599. doi: 10.2196/48599 (PMC10865187; doi:10.2196/48599)
Supplement: Multimedia Appendix 2 [file jmir_v26i1e48599_app2.docx]

**Supplementary Material II**


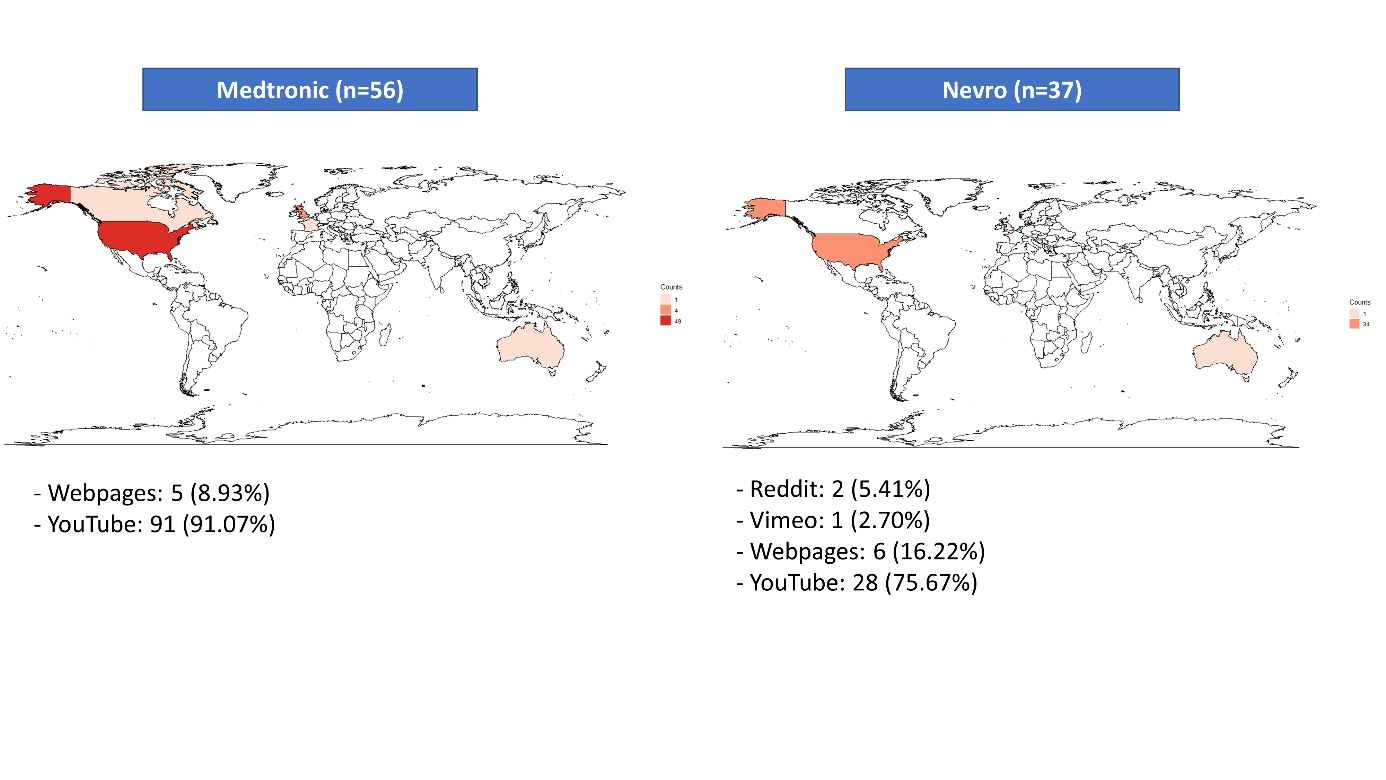


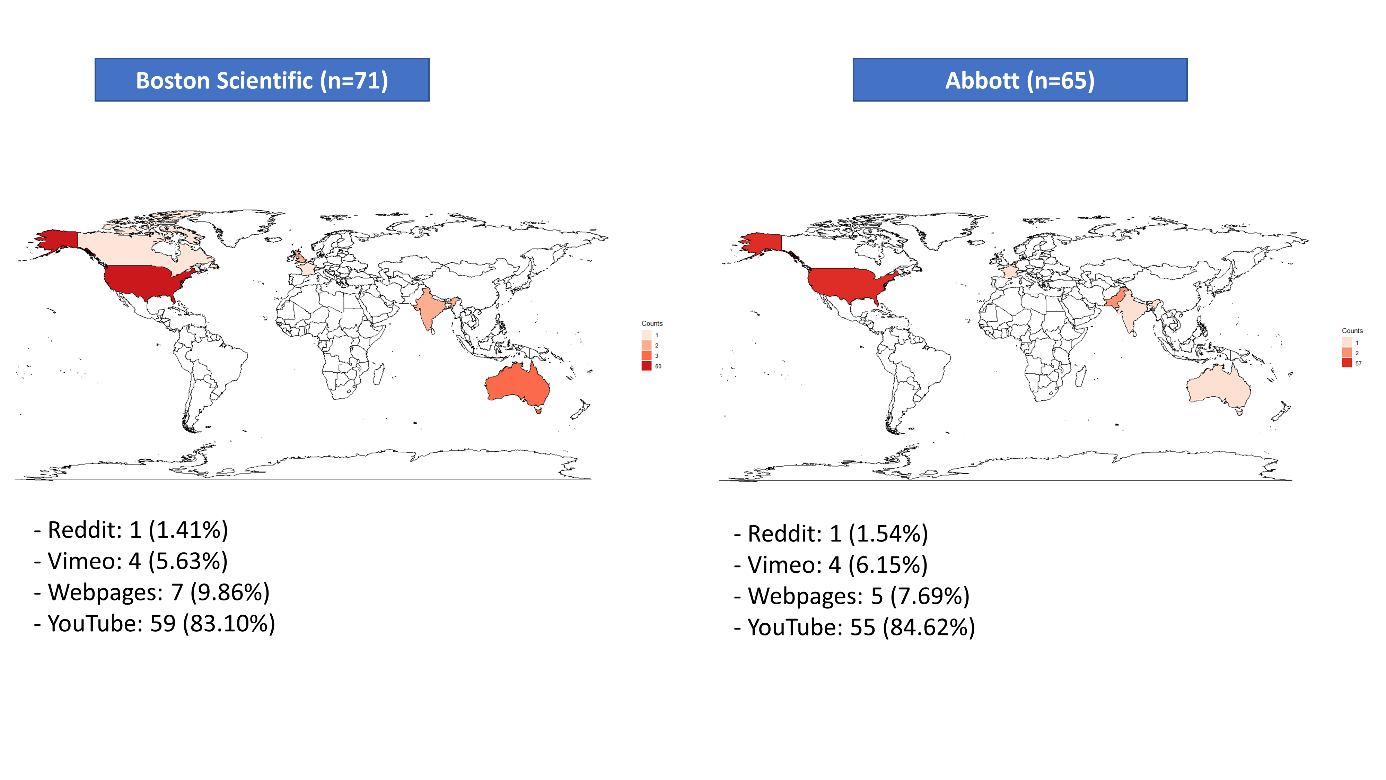


World map and type of entries separated by prominent industrial companies of neuromodulation devices.

| Category | Question | Medtronic | Nevro | Boston Scientific | Abbott (incl. St. Jude Medical) |
| --- | --- | --- | --- | --- | --- |
| Reliability | Are the aims clear? | 1.6 | 1.5 | 1.6 | 1.4 |
|  | Does it achieve its aims? | 3.2 | 3.1 | 2.5 | 3.5 |
|  | Is it relevant? | 3.3 | 3.1 | 3.0 | 2.9 |
|  | Is it clear what sources of information were used to compile the publication? | 2.8 | 3.2 | 3.3 | 3.0 |
|  | Is it clear when the information used or reported in the publication was produced? | 2.9 | 3.2 | 3.2 | 3.1 |
|  | Is it balanced and unbiased? | 3.9 | 3.3 | 3.7 | 3.5 |
|  | Does it provide details of additional sources of support and information? | 2.8 | 2.6 | 2.6 | 2.3 |
|  | Does it refer to areas of uncertainty? | 2.3 | 2.8 | 2.7 | 2.3 |
| Content of information | Does it describe how each treatment works? | 3.2 | 3.2 | 2.9 | 3.2 |
|  | Does it describe the benefits of each treatment? | 2.6 | 2.4 | 2.1 | 2.3 |
|  | Does it describe the risks of each treatment? | 2.5 | 1.6 | 1.5 | 1.7 |
|  | Does it describe what would happen if no treatment is used? | 1.1 | 1.2 | 1.2 | 1.2 |
|  | Does it describe how the treatment choices affect overall quality of life? | 2.7 | 3.1 | 3.3 | 2.9 |
|  | Is it clear that there may be more than one possible treatment choice? | 1.7 | 1.9 | 2.2 | 2.0 |
|  | Does it provide support for shared decision-making? | 3.1 | 2.3 | 2.4 | 2.5 |
| overall quality | Based on the answers to all of the above questions, rate the overall quality of the publication as a source of information about treatment choices | 2.5 | 2.4 | 2.5 | 2.4 |

Scoring of quality appraisal with the DISCERN instrument, separated by prominent industrial companies of neuromodulation devices.
