# Supplementary figures and images for: Examining the Type, Quality, and Content of Web-Based Information for People With Chronic Pain Interested in Spinal Cord Stimulation: Social Listening Study
Source: J Med Internet Res. 2024 Jan 30;26:e48599. doi: 10.2196/48599 (PMC10865187; doi:10.2196/48599)

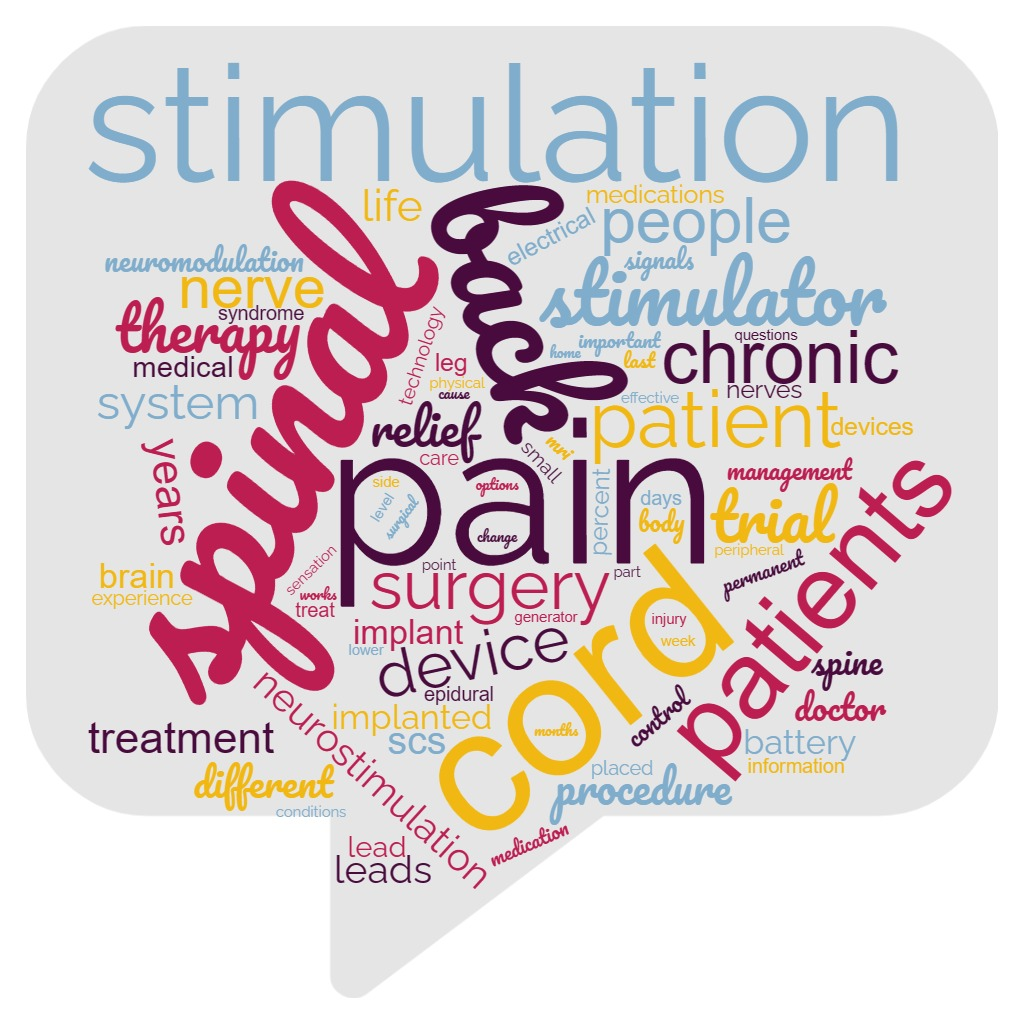

Supplement: Multimedia Appendix 3 [file jmir_v26i1e48599_app3.png]
